# Adaptively Tuned Iterative Low Dose CT Image Denoising

**DOI:** 10.1155/2015/638568

**Published:** 2015-05-24

**Authors:** SayedMasoud Hashemi, Narinder S. Paul, Soosan Beheshti, Richard S. C. Cobbold

**Affiliations:** ^1^Institute of Biomaterials and Biomedical Engineering, University of Toronto, Toronto, ON, Canada M5S 3G9; ^2^Joint Department of Medical Imaging, Toronto General Hospital, University Health Network, Toronto, ON, Canada M5G 2N2; ^3^Department of Electrical and Computer Engineering, Ryerson University, Toronto, ON, Canada M5B 2K3

## Abstract

Improving image quality is a critical objective in low dose computed tomography (CT) imaging and is the primary focus of CT image denoising. State-of-the-art CT denoising algorithms are mainly based on iterative minimization of an objective function, in which the performance is controlled by regularization parameters. To achieve the best results, these should be chosen carefully. However, the parameter selection is typically performed in an ad hoc manner, which can cause the algorithms to converge slowly or become trapped in a local minimum. To overcome these issues a noise confidence region evaluation (NCRE) method is used, which evaluates the denoising residuals iteratively and compares their statistics with those produced by additive noise. It then updates the parameters at the end of each iteration to achieve a better match to the noise statistics. By combining NCRE with the fundamentals of block matching and 3D filtering (BM3D) approach, a new iterative CT image denoising method is proposed. It is shown that this new denoising method improves the BM3D performance in terms of both the mean square error and a structural similarity index. Moreover, simulations and patient results show that this method preserves the clinically important details of low dose CT images together with a substantial noise reduction.

## 1. Introduction

While X-ray computed tomography (CT) enables ultrafast acquisition of patient images obtained with excellent spatial resolution, the dose needed to achieve diagnostic image quality can result in a significant increase in the risk of developing cancer [1]. Consequently, low-dose CT imaging is clinically desired and has been under investigation for several years. Lowering the radiation dose may seriously degrade diagnostic performance or undermine physician confidence by producing noisier images [2, 3]. Several different algorithmic approaches have been proposed to reduce the effect of noise in the CT images, including projection data denoising [4–6], optimizing the reconstruction algorithms to include the noise statistics [7–9], and CT image denoising [10–12]. The latter is the focus of this paper, where an adaptively tuned iterative CT image denoising algorithm is presented.

The main source of noise in X-ray projection data is quantum noise caused by statistical fluctuations of X-ray quanta reaching the detectors, so that the CT projection noise follows the Poisson distribution [3]. However, because of the use of different reconstruction algorithms and signal processing steps in CT reconstruction, the noise statistics of the processed CT images are usually unknown and hard to model and are spatially changing. Moreover, directional noise in the form of streak artifacts is present in many CT images. As a result, incorporating accurate noise statistics into image-based CT denoising can be very challenging. When denoising is based on the projection data and its statistics, other difficulties arise. Specifically, such denoising methods and the associated iterative reconstructions require access to the CT raw data, which is often unavailable. Furthermore, these methods have a high computational complexity making it challenging to obtain a final image in a reasonable length of time, depending on the available computational resources. On the other hand, image-based denoising methods are fast and can be applied directly on the CT images without changing the clinical workflow.

A simplified noise model is usually used in image based denoising algorithms, in which, following the Central Limit Theorem (CLT) [13], the final noise in each voxel follows a Gaussian distribution [11, 14–17]. The CLT can be used since each voxel in CT images is computed by adding values from many different projections. With this assumption, a noisy CT image **y** can be modeled by

(1) $$\begin{array}{c} y=x+n, \end{array}$$

where **x** is the noiseless image and **n** is a zero mean additive anisotropic Gaussian noise with variance of *σ*_*n*_^2^, which varies with the pixel location and its value.

Different image based denoising algorithms have been used to estimate the noiseless CT images, such as anisotropic diffusion [10], total variation (TV) [18], bilateral filtering [19], or wavelet-based techniques [11, 12, 20]. These methods can usually be formulated as an unconstrained Lagrangian multiplier optimization problem [18, 21–23], that is,

(2) $$\begin{array}{c} \hat{x}=\underset{x}{\arg\;\min}\frac{1}{2}{\left\|\;x-y\;\right\|}_{2}^{2}+\lambda h\left(\;x\;\right), \end{array}$$

in which *λ* is a regularization parameter that controls the tradeoff between the data fidelity and the regularization term *h*(*x*) and ‖*x*‖_*q*_ = ∑_*i*=1_^*n*^|*x*_*i*_|^*q*^. Different regularization terms, *h*(*x*), lead to different denoising methods. For example, in TV-based methods [24–26], $h(x)=\sqrt{\delta_{h}x^{2}+\delta_{v}x^{2}}$ where *δ*_*h*_ and *δ*_*v*_ are the gradient in horizontal and vertical directions, and in wavelet soft thresholding methods *h*(*x*) = ∑_*i*_ | Ψ_2D_ *x*)_*i*_|, where Ψ_2D_(*x*) is the 2D wavelet transform [27].

There is a strong dependence of the quality of the result on the regularization parameter. It is a challenging task to find the regularization parameter *λ* that provides the best balance between signal smoothing and feature preservation [26]. Specifically, if *λ* is not appropriately adjusted, the optimization is trapped in a local minimum; that is, if *λ* is too small, noise is only partially removed and, if it is too large, the image may be oversmoothed [25]. Some methods have been proposed to update the regularization parameters iteratively, such as use of the discrepancy principle [28], generalized cross-validation [26], and L-curve [29]. These methods fail in certain situations, are problem specific, and generally increase the computational complexity of the algorithms.

One straight forward approach used in many algorithms is to use a heuristic *λ* value combined with a criterion to stop the algorithm before the estimated signal is oversmoothed. Different stopping criteria have been proposed for iterative denoising problems. For instance, Akkoul et al. [30] used a switching median filter algorithm to stop the iterative process when the number of changed pixels in the denoising iterations is a minimum. In [20, 31] the statistical properties of high frequency wavelet subbands were used to stop the TV iterations. However, such methods are unable to differentiate oversmoothed data from well-denoised data. As a result, to avoid oversmoothing, the updating steps are typically chosen to be small, which decreases the convergence speed.

In this paper, a noise confidence region evaluation (NCRE) method is used to address the regularization selection and the algorithm stopping problems. It adaptively updates the regularization parameters at the end of each iteration by validating the result of that iteration. The algorithm stops when the statistical properties of the denoising residual resemble those of the additive white Gaussian noise. Using NCRE, a new iterative block matching and 3D filtering (BM3D) method is proposed, which outperforms BM3D [32] itself. The proposed method is compared with anisotropic diffusion denoising, which is generally regarded as a standard denoising method in CT imaging [10], nonlocal mean [33–35], and BM3D [32]. In addition, we study the noise properties of CT images and show that the noise in small image blocks has an additive white Gaussian model, which justifies the success of the nonlocal based denoising algorithms in image based CT denoising [33, 36–39].

## 2. Problem Formulation and CT Noise Properties

Recently, it has been shown that nonlocal patch based algorithms outperform others in CT image denoising [33, 34, 36–41]. For example, in [41] a nonlocal means (NLM) based method, which takes advantage of the presence of repeating structures in a given image, was compared with a principle component analysis based denoising method and a highly constrained backprojection method. It was shown that the NLM method outperformed both of the methods in terms of the contrast to noise ratio, noise standard deviation, and squared error. Another class of algorithms looks for similar blocks in the whole 2D image and stacks them together in 3D arrays. Denoising is then performed through transform domain shrinkage of the 3D arrays. An algorithm called K-SVD [37, 38, 42] uses these 3D patches to train an optimum dictionary. This method, which assumes that each 3D block can sparsely be represented by the trained dictionary atoms, uses shrinkage algorithms to denoise the patches.

Our proposed algorithm makes use of the block matching and 3D filtering (BM3D) technique [32, 36]. This is a noniterative denoising method that currently outperforms many newer algorithms [40]. It is composed of two major filtering steps. In both stages collaborative filtering is utilized, which itself has four stages: (1) grouping similar patches with a reference patch, (2) calculation of the 3D wavelet coefficients of each stack of patches, (3) denoising the wavelet coefficients (thresholding in step (1) or Wiener filtering in step (2)), and (4) recovering the denoised image by calculating the inverse 3D wavelet transformation. The BM3D approach aims to denoise the patches by Wiener filtering, which is done in step (2). To find best match to similar patches in the step (2) and to reliably estimate the Wiener coefficients, the method requires a reliable estimate of the noiseless image, which is the main purpose of step (1). The input for this step, which is a hard thresholding block, is the 3D noisy wavelet coefficients of similar patches located by block matching applied to the available noisy image. A hard threshold with a heuristically determined value of 2.7*σ*_*n*_ is used in step (1). The resulting denoised coefficients are then transformed back to a spatial domain to be used as the initial estimate of the noiseless data used for calculating the Wiener filter coefficients.

To denoise the images, patch based methods generally use a model based on

(3) $$\begin{array}{c} y_{G_{j}}=x_{G_{j}}+n_{G_{j}},\;j\in \left[1,\ldots ,m\right], \end{array}$$

in which *G* is the patch grouping information, *m* is the number of 3D patches, **y**_*G*_*j*__ denotes the noisy patches, **x**_*G*_*j*__ denotes the noiseless 3D patches, and **n**_*G*_*j*__ ~ *𝒩*(0, *σ*_*G*_*i*__^2^) is the noise at each 3D patch. Conventional BM3D uses an independent identical additive Gaussian noise model in which the noise variances *σ*_*G*_*i*__^2^ are similar in all patches. Using this assumption, its regularization term is a nonlocal wavelet *ℓ*_1_-norm *h*(*x*) = NLW(*x*, *G*), where [43]

(4) $$\begin{array}{c} \text{NLW}\left(\;x,G\;\right)=\overset{m}{\underset{j=1}{\sum }}{\left\|\;\Psi_{\text{3D}}\left(\;x_{G_{j}}\;\right)\;\right\|}_{1}, \end{array}$$

in which Ψ_3D_ is the 3D wavelet transform. Using this regularization in (2), BM3D solves the following optimization problem in its first step:

(5) $$\begin{array}{c} \hat{x}=\underset{x}{\arg\;\min}\frac{1}{2}{\left\|\;x-y\;\right\|}_{2}^{2}+\lambda \overset{m}{\underset{j=1}{\sum }}{\left\|\;\Psi_{\text{3D}}\left(\;x_{G_{j}}\;\right)\;\right\|}_{1}. \end{array}$$

In our approach, we modify the BM3D formulation for CT image denoising by incorporating a more realistic noise model, to include the nonstationarity of the noise and its dependence on the position and value of the pixels. Our proposed method uses the noise properties of the patches **x**_*G*_*j*__, studied in the Appendix, to improve the performance of BM3D for CT image denoising.

### 2.1. Noise in CT Images

Although a reasonable statistical model for the CT projection data is the independent Poisson distributions [3], it has been shown that the corrected polyenergetic X-ray projections can be modeled more accurately by a Gaussian distribution with the following relationship between its mean and variance:

(6) $$\begin{array}{c} \sigma_{i,n}^{2}=\sqrt{{\left(J_{i}\times \exp\left(\frac{\bar{\xi }\left(i,n\right)}{s}\right)\right)}^{2}+{\left(\sigma_{e}^{2}\right)}_{i,j}}, \end{array}$$

where $\bar{\xi }$ is the mean and *σ*_*i*,*n*_^2^ is the variance of the projections at the *i*th projection angle (*φ*_*i*_) and the *n*th detector bin whose distance is *l*_*n*_ from the detectors center, *s* is a scaling factor, *σ*_*e*_^2^ is the electronic noise variance, and *J*_*i*_ is a parameter adaptive to different detector channels [6]. During the reconstruction process the noise distribution is changed by the reconstruction algorithm and filters. As a result, due to the complicated dependencies of noise on scan parameters and on spatial position, the noise distribution in the reconstructed CT images is usually very difficult to determine (a detailed study of the noise model in CT can be found in [44, 45]). Using the discrete filtered back projection relation,

(7) $$\begin{array}{c} f\left(\;x,y\;\right)=\frac{\pi \Delta t}{N_{\pi g}}\overset{N_{\pi g}}{\underset{i=1}{\sum }}\overset{N_{g}}{\underset{n=1}{\sum }}c\left(\;x\cos\varphi_{i}-y\sin\varphi_{i}-l_{n}\;\right)g_{i,n}, \end{array}$$

the noise variance in the reconstructed images can be described by [38]

(8) σn2x,y=πΔtNπg2∑i=1Nπg∑n=1Ngc2xcos⁡φi−ysin⁡φi−lnσi,n2,

where Δ*t* is the distance between the center of two adjacent detectors, *g*_*i*,*n*_ is the parallel projection at the *i*th angle and the *n*th detector bin, and *c*(·) is the ramp filter in the spatial domain. This can be interpreted as the backprojection of the projection noise variances, making it nonstationary, object dependent, and correlated. Moreover, since the variance of each voxel is the summation of the variances from many angles, if the variance in one direction is significantly larger than that at another direction, then the variances along that direction will be more correlated than that for other directions [46] producing what is known as a streak artifact in the reconstructed images. It should be noted that this effect is not included in our model. In (8), the ramp filter *c*(·) is symmetric about the center of rotation and *σ*_*i*,*n*_^2^ depends on the attenuation of the media through which the X-ray beams pass. Therefore, we assert that the noise variances of small neighborhoods with similar attenuations and similar radial distances from the center of rotation should be very similar. The results of experimental tests of this assertion are presented in the Appendix.

### 2.2. Modified Formulation

Based on the above discussion and our experimental results, presented in the Appendix, it can be assumed that the noise in 3D similar patches of the CT images follows a white additive Gaussian distribution, with different variances for different 3D patches *σ*_*G*_*i*__^2^. Using (3), the modified optimization problem used in this paper is given by

(9) Jy,G,Λx^=arg minx⁡12x−y22+∑j=1mλjΨ3DxGj1,

in which Λ = [*λ*_1_, *λ*_2_,…, *λ*_*m*_] is a set of regularization parameters that are functions of *σ*_*G*_*i*__. To improve the regularization parameter selection, we used an adaptive updating method based on an evaluation of the noise statistics. It automatically avoids the oversmoothing without lowering the convergence speed. The NCRE method validates the statistical properties of the error residuals at the end of each iteration and categorizes the result as well denoised, partially denoised, or oversmoothed. This information is then used to update the parameters in the next iteration or to stop the algorithm at the end of the iteration for which the similarity between error residual and Gaussian noise is satisfied.

## 3. Proposed Denoising Method: BM3D-NCRE

If ${\hat{x}}_{i}$ denotes the signal recovered at *i*th iteration, the denoising residual at the end of *i*th iteration can be expressed by $\Delta y_{i}=y-{\hat{x}}_{i}$ which, ideally, should be the noise (3), **n** = {**n**_*G*_*i*__, ∀*i* = 1,…, *m*}. Here, we provide a quantitative measure that verifies the similarity between the structure of Δ**y**_*i*_ and that of **n**.

### 3.1. Noise Confidence Region Evaluation (NCRE)

In [47] it was shown that the following function of zero mean white Gaussian noise, **n**, with length *n*, and for any given scalar value of *z*:

(10) Gz,n=1n∑j=1nGz,nj,Gz,nj=1if  nj≤z0if  nj>z;

is equivalent to sorting the absolute value of the noise elements **n**_*j*_. The expected value of this function is *E*(*𝒢*(*z*, **n**)) = *F*(*z*) and its variance is var(*𝒢*(*z*, **n**)) = *F*(*z*)(1 − *F*(*z*))/*n*, where *F*(*z*) = 2*ϕ*(*z*/*σ*_*n*_) − 1 and *ϕ*(·) is the cumulative distribution function (CDF) of a Gaussian distribution. Therefore, *𝒢*(*z*, **n**) is bounded by the following lower (*L*_*n*_) and upper (*U*_*n*_) values:

(11) LnzFz−ζ1nFz1−Fz,Unz=Fz+ζ1nFz1−Fz

with probability of $Pr\{|𝒢(z,n)-F(z)|\leq \zeta \sqrt{\left(1/m\right)F(z)(1-F(z))}\}\approx 2\phi (\zeta )-1$. If the sorted absolute values of a signal lie between these two boundaries for a large enough *ζ*, that signal will follow a white Gaussian distribution with a confidence probability close to one.

**Figure 1:**
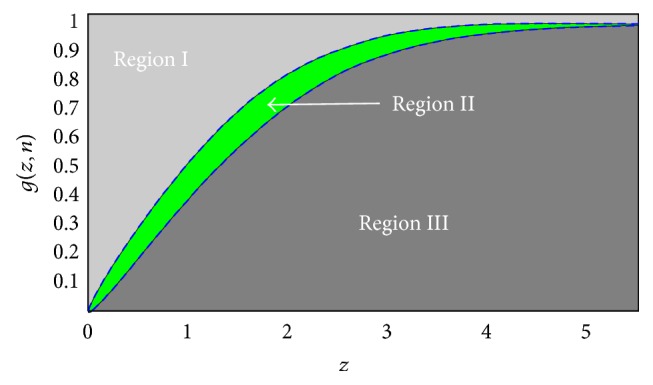
Three possible regions for the residual at the end of each iteration. If it lies in Region II (noise confidence region), denoising is stopped.

**Figure 2:**
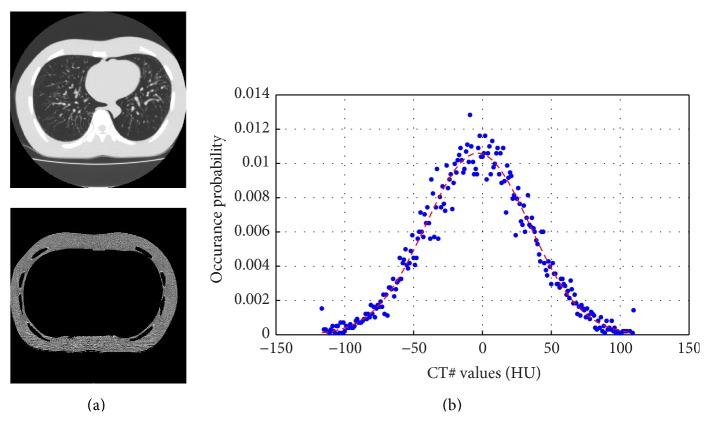
Noise statistics of the soft tissue region surrounding the lung. (a) Top: the thoracic phantom which is used to evaluate the noise characteristics and bottom: the soft tissue region of the phantom (denoted by *ℳ* in Algorithm 1). (b) The statistical distribution of the noise in the soft tissue region is shown by the blue experimental values and these are compared with Gaussian distribution with the same variance (red dashed line).

As shown in Figure 1, these boundaries divide the [*z*, *𝒢*(*z*, **n**)] space into three regions. At the end of each iteration *𝒢*(*z*, Δ**y**_*i*_) the sorted absolute value of the residual is calculated. If this sequence falls into Region II (in our proposed algorithm being a subset of a region is evaluated by having a high fraction of *𝒢*(*z*, Δ**y**_*i*_) in that region; for example, in our simulations this fraction is 90%), it means that the residual has a Gaussian-like structure and denoising stops. On the other hand, if the denoising at the *i*th iteration has removed not only the noise but also parts of the noiseless data itself, Δ**y**_*i*_ will have some of the image information making its samples larger than Gaussian noise. Therefore, for a specific value of *z*, *𝒢*(*z*, Δ**y**_*i*_) (average number of Δ**y**_*i*_s with absolute values smaller than *z*) is smaller than *𝒢*(*z*, *n*) and falls in Region III. This will enforce continuation of the denoising to the (*i* + 1)th step with changing of the regularization parameters *λ*_*j*_ such that the denoising algorithm extracts less noise in the next iteration, that is,* decreasing *∀*λ*_*j*_ = *λ*_*j*_/*s*, *s* > 1. If *𝒢*(*z*, Δ**y**_*i*_) falls in Region I, it indicates that the noise is partially removed. In this case the algorithm continues to the (*i* + 1)th step and changes the regularization parameters *λ*_*j*_ such that more noise is extracted by the denoising algorithm, that is,* increasing *∀*λ*_*j*_ = *s* × *λ*_*j*_, *s* > 1. In summary, at each iteration when *𝒢*(*z*, Δ**y**_*i*_) is in either Region I or III, the regularization parameter is updated such that it moves toward Region II. The value of *s* can be tuned as a fixed or an adaptively changing variable based on the euclidean distance between *𝒢*(*z*, Δ**y**_*i*_) and *F*(*z*), *d*_*i*_ = ‖*𝒢*(*z*, Δ**y**_*i*_) − *F*(*z*)‖^2^. In our proposed method to update Λ = [*λ*_1_,…, *λ*_*m*_] a global *s* is used (similar to [33, 41]), which is updated based on the placement of the denoising residual of the patches in different Regions I–III. The algorithm is stopped when the denoising residual of the soft tissue around the lung is placed in Region II. An example of a soft tissue mask for a thoracic phantom, denoted by *ℳ*, is shown in Figure 2. The pixels in this region have very similar CT# and have almost the same radial distances from the center of the rotation. Therefore, it could be assumed that the noise in this region has a white Gaussian distribution.

### 3.2. Summary of the Proposed Method: BM3D-NCRE

Algorithm 1 shows the proposed iterative Λ updating scheme, in which · denotes an element-wise multiplication. The updating method uses a memory strategy for recovery of possible lost edges and fine details. This process is represented by

(12) $$\begin{array}{c} y_{i}=\left(\;1-\alpha_{k}\;\right)y+\beta_{k}{\hat{x}}_{i}+\left(\;\alpha_{k}-\beta_{k}\;\right){\hat{x}}_{i-1}, \end{array}$$

in which *k* ∈ [1,2] and *α*_*k*_ and *β*_*k*_ are positive scalars, chosen based on the conditions given in [48]. This stage of the algorithm was inspired by the second order iterative methods [48, 49] that improve the convergence rate of the iterative methods.

To denoise CT images the fundamentals of BM3D were used in an iterative scheme: the output of the Wiener filter is a better estimate of the original image than the input of Wiener filter from the first step. Therefore, this output can be fed into the first step to provide better Wiener coefficients in the second iteration. The modified BM3D formulation (9) is used iteratively in Algorithm 1 to denoise the CT images, where NCRE adjusts the threshold values applied on 3D wavelet coefficients of the first step. The parameters of the BM3D algorithm are chosen based on the ones that resulted in the best performance in [50]. The initial value for Λ is 2.7*σ*_*G*_*i*__ with noise variances estimated independently for each 3D stack, using the median-absolute deviation method as described in [51]. In each iteration, if the sorted absolute value of the denoising residual of the soft tissues around the lung, *ℳ* · Δ**y**_*i*_, falls into Region I, the threshold value will be increased and, if it falls into Region III, the threshold values are decreased, so that in the next iteration the residual moves towards Region II.

## 4. Results and Discussion

Three test methods were used to evaluate the performance of the proposed algorithm. The first method consisted of a simulated Shepp-Logan phantom corrupted by adding Poisson noise to the fan beam X-ray projections. The images were reconstructed using the ifanbeam command in Matlab. The number of unattenuated photons in each projection was taken to be 1 × 10^13^, which led to a noise variance similar to the images reconstructed from the tube current of 50 mAs and peak voltage of 120 kVp. White Gaussian noise with standard deviation of 100 was added to all the projections to simulate the presence of electronic noise. The second method used a CATPHAN phantom (Phantom Laboratory, Greenwich, NY, USA). This is a standard phantom widely used for CT image quality evaluation and contains spheres of differing contrast as well as line pairs with differing spacing that can be used to test the spatial resolution. Ideally, the denoising algorithms should enable us to distinguish between smaller spheres with lower contrasts in the low contrast slice and should keep the line pair resolution unchanged. The third method uses axial chest CT images from a clinical patient. All three test methods used the following parameters in the Algorithm 1: *ζ* = 6, *α*_1_ = 0.9, *β*_1_ = 0.8, *α*_2_ = 0.4, and *β*_2_ = 0.3. The parameters of BM3D are chosen similar to the ones proposed in [50]: the size of the blocks is 8 × 8, sliding step to process every next reference block is 3, maximum number of similar blocks is 16, and the size of the search neighborhood for full-search blockmatching is 39 × 39.

**Figure 3:**
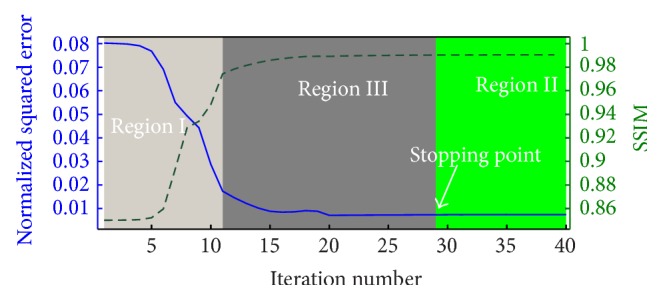
Squared error (blue line) and the Structural Similarity Index (SSIM) (green dashed line) showing the changes with each iteration when using BM3D-NCRE. The shading colors show the region in which Δ**y**_*i*_'s are placed after each iteration. The algorithm stops when Region II is reached.

**Figure 4:**
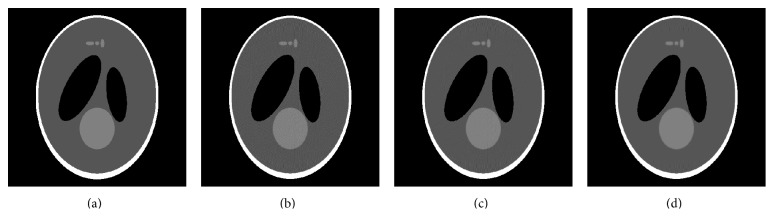
Shepp-Logan phantom simulations: (a) original phantom, (b) noisy reconstructed phantom, (c) denoised by BM3D, and (d) denoised by BM3D-NCRE.

Figure 3 shows the mean square error (MSE) and the structural similarity index (SSIM) [52] resulting from successive iterations of BM3D-NCRE applied to the reconstructed noisy Shepp-Logan phantom images. The first iteration is equivalent to the result of BM3D. As shown, the MSE decreases and the SSIM increases in successive iterations to a point where the algorithm is stopped by falling into Region II. The results of denoising the Shepp-Logan phantom with BM3D and BM3D-NCRE are shown in Figure 4. As can be seen, the noise is removed more effectively by BM3D-NCRE. However, the streak artifacts are still visible in both denoised images.

**Figure 5:**
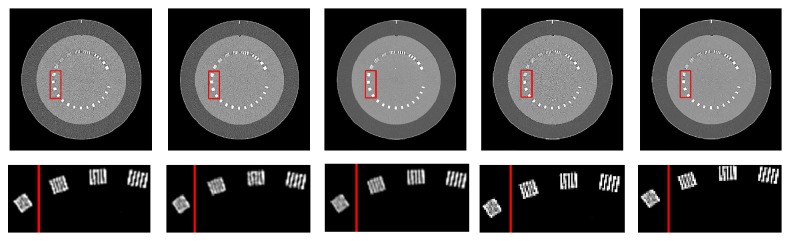
Top: line pair slice of the CATPHAN phantom scanned with 50 mAs and 120 kVp (window width/window level = 400/60 HU). Bottom: red rectangular ROI of the images with the red line showing the cut-off line pair resolution (window width/window level = 400/500 HU). Left to right: image reconstructed with FC52 (STD = 64 HU) and reconstructed with AIDR3D (STD = 41 HU), FC52 denoised with the proposed method (STD = 22 HU), FC52 denoised with nonlocal mean (STD = 34 HU), and FC52 denoised with BM3D method (STD = 27 HU).

In the second test, the CATPHAN phantom was scanned using a low dose (50 mAs, 120 kVp) and a high dose (300 mAs, 120 kVp) protocol. Image reconstructions were performed with a Toshiba Aquilion One CT using the proprietary lung kernel FC52 and the proprietary iterative reconstruction algorithm Adaptive Iterative Dose Reduction 3D (AIDR3D) [53]. The latter uses anisotropic diffusion denoising as its base to improve the image quality at each iteration. Our proposed denoising method was applied to the images reconstructed with the high spatial resolution filter algorithm, FC52. These are compared to the images reconstructed with AIDR3D, and the FC52 reconstructed images denoised by nonlocal mean and BM3D. The nonlocal mean package provided by Gabriel Peyre on Mathwork File Exchange was used here (http://www.mathworks.com/matlabcentral/fileexchange/13619-toolbox-non-local-means). This package is based on the method described in [35]. The BM3D code is also based on the package provided by Alessandro Foi on his homepage (http://www.cs.tut.fi/~foi/GCF-BM3D/). It should be noted that the parameters of nonlocal mean and BM3D were heuristically adjusted to achieve the best performance, based on visual inspection of the results. In addition, the parameters are adjusted to keep the spatial resolution in the line pair resolution slice the same.  Figure 5 shows the line pair slice reconstructed by FC52, AIDR3D, and FC52 denoised by BM3D-NCRE, nonlocal mean, and BM3D. As can be seen, all these methods have the same spatial resolution as the original image whose resolution was not improved by the proprietary iterative reconstruction method used (AIDR3D).

**Figure 6:**
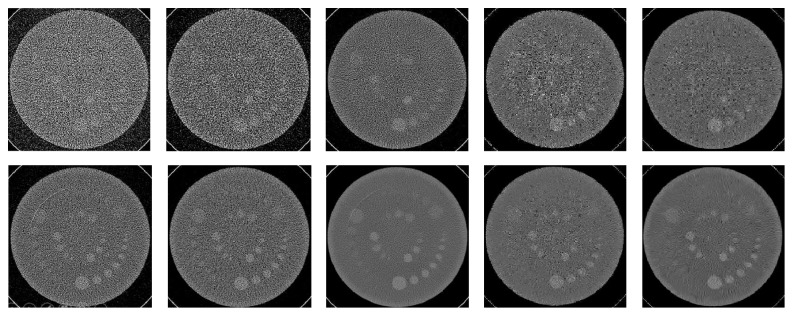
Low contrast study using CATPHAN low contrast slice. Top: images scanned with 50 mAs/120 kVp and bottom: scanned with 300 mAs/120 kVp. Left to right: image reconstructed with FC52, reconstructed with AIDR3D, reconstructed with FC52 and denoised by BM3D-NCRE, reconstructed with FC52 and denoised by nonlocal mean, and reconstructed with FC52 and denoised by BM3D. In all images window width/window level = 100/70 HU.

Figure 6 shows that the detectability of low contrast objects is improved with our method and outperforms that achieved by AIDR3D, nonlocal mean, and BM3D. Visibility of the spheres with higher contrasts in the images denoised by BM3D is very close to the ones denoised by BM3D-NCRE. However, the visibility is significantly less for the spheres with lower contrasts. Both BM3D and BM3D-NCRE outperform AIDR3D and nonlocal mean, while the number of visible spheres is almost the same as in the images denoised by nonlocal mean and the images reconstructed by AIDR3D.

**Figure 7:**
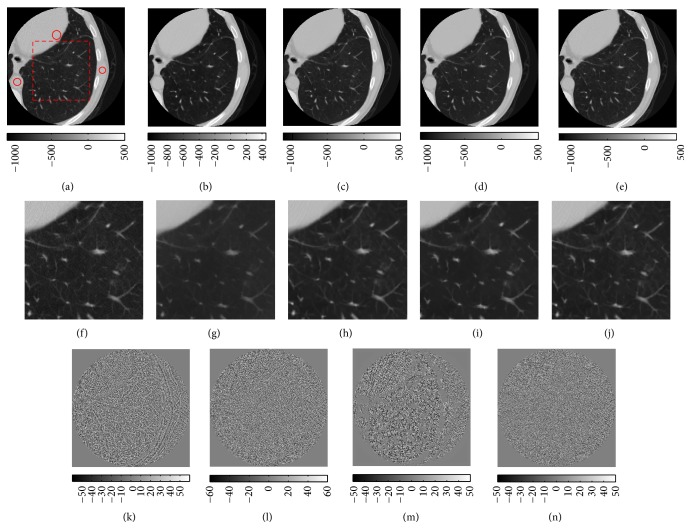
Comparison of the effects of anisotropic diffusion, nonlocal mean, BM3D, and BM3D-NCRE. (a) In the original image, the circular regions show the area from which the noise variance is measured, the dashed rectangular region is the area which is shown in (d)-(e), and average noise of the three regions is around 55 HU. (b) Denoised by anisotropic diffusion, noise is around 25 HU. (c) Denoised by BM3D-NCRE, noise is around 25 HU. (d) Denoised by nonlocal mean, noise is around 21 HU. (e) Denoised by BM3D, noise is around 28 HU. ((f)–(j)) The zoomed-in region shown by dashed rectangle in image (a). The difference between original image and the image denoised by (k) anisotropic diffusion, (l) BM3D-NCRE, (m) nonlocal mean, and (n) BM3d are shown. The window width/window level in (a)–(j) is 1600/−300 HU and is 100/0 HU in (k)–(n).

The final comparison was made using a low dose (50 mAs, 120 kVp) lung CT of a patient reconstructed using FC52 and processed by anisotropic diffusion denoising, BM3D-NCRE, nonlocal mean, and BM3D. A single axial slice of the images is shown in Figure 7. As can be seen, anisotropic diffusion removes some fine details and reduces the contrast of the small features. Nonlocal mean, BM3D, and BM3D-NCRE outperform anisotropic diffusion denoising in sense of preserving the small structures. Comparing the results of nonlocal mean with BM3D-NCRE, it can be seen that the low contrast features are kept perfectly unchanged in BM3D-NCRE, while they are removed or their contrasts are reduced in the image denoised by nonlocal mean. In addition, the noise is not homogeneously removed from the image. Comparing BM3D with BM3D-NCRE, it can be seen that the image denoised by BM3D-NCRE has less noise and the removed noise is more homogeneous. It should be noted that the parameters of the anisotropic diffusion denoising are adjusted in such a way that the noise variance in the reconstructed images is the same as the images denoised by BM3D-NCRE.

## 5. Conclusions

An iterative denoising scheme was proposed for low dose CT images, which adjusts the denoising parameters at each iteration based on the effect of the denoising method in the previous iteration. Noise confidence region evaluation (NCRE) was used to compare the Gaussian noise with denoising residual to determine if the denoising was effectively, weakly, or strongly executed. Based on this information the denoising parameters were adjusted for the next iteration. BM3D was used in the new proposed iterative scheme. The phantom study showed that our proposed method improved low contrast detectability. The patient study demonstrated that the image was efficiently denoised and the visibility of small objects was preserved. However, it should be noted that the modified optimization model is not accurate when the electronic noise dominates the photon fluctuations as could occur for very low doses. In addition, the streak artifacts would still be present in images denoised by the proposed method.

## Figures and Tables

**Figure 8:**
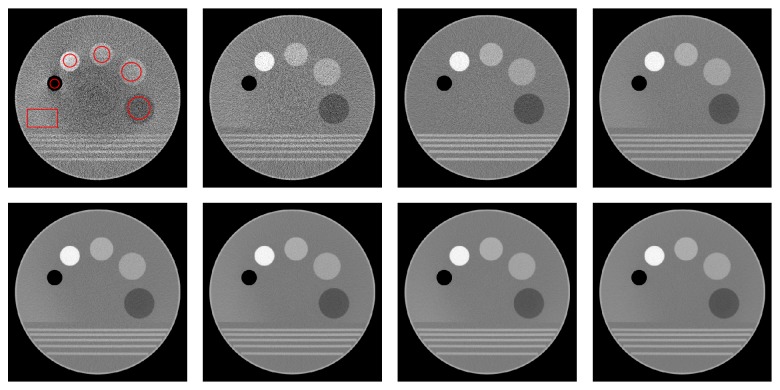
Phantom scanned with eight different X-ray source currents with the same peak voltage: top left to right 5, 10, 25, and 50 mAs and bottom left to right 100, 150, 200, and 250 mAs.

**Figure 9:**
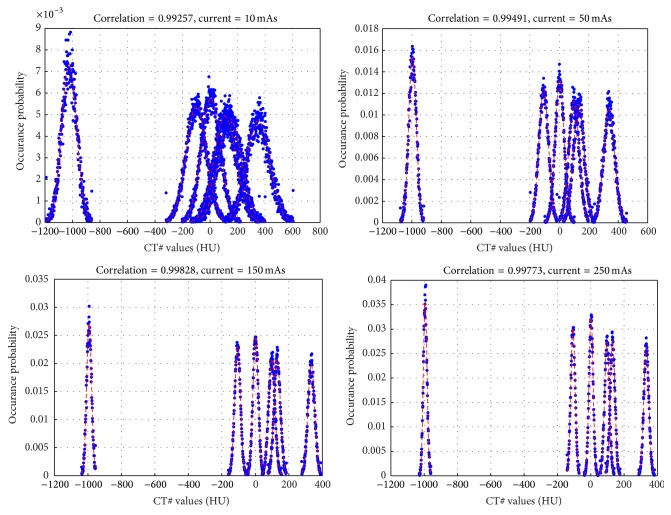
Comparison of the noise distribution in the six regions of the phantom shown in Figure 8 with white Gaussian noise. Blue dots are the measured values and the red dashed lines are the fitted Gaussian distributions.

**Figure 10:**
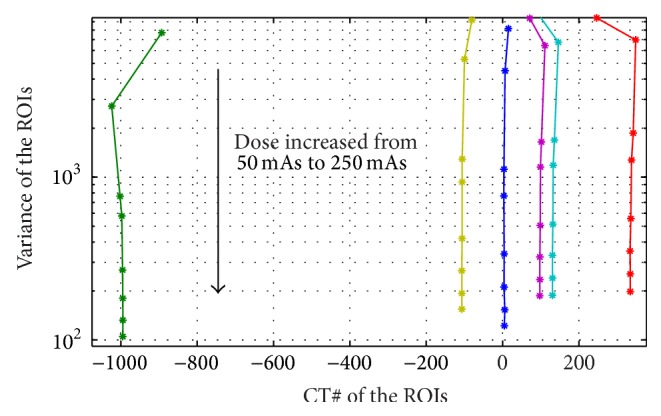
Showing the noise variance changes for the background and the five circular regions of the phantom as the dose was increased from 5 mAs to 250 mAs.

**Figure 11:**
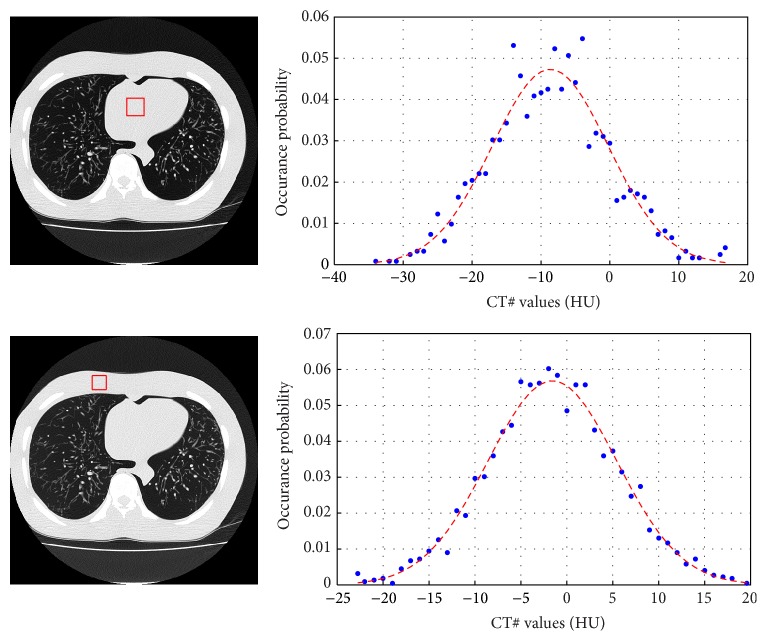
Two small soft tissue regions in the lung of a thoracic anthropomorphic phantom are shown. The noise distribution of these regions (blue dots), which could be used in patch based denoising, is compared to that of the fitted Gaussian distribution (dashed red lines).

**Algorithm 1:**
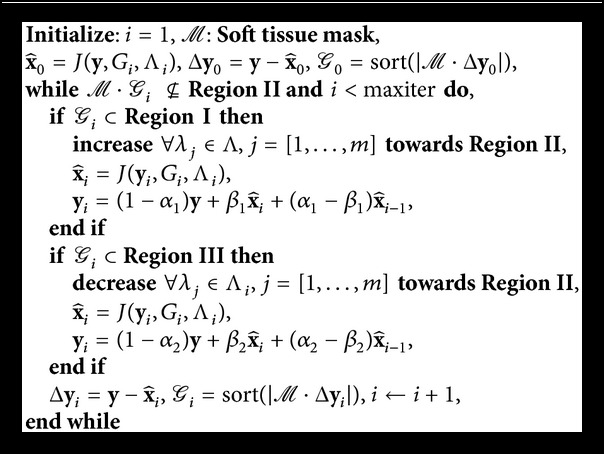
Proposed Iterative Regularization Parameter Updating.

## Appendix

### Phantom Study of the Noise Statistics in Reconstructed CT Images

The noise statistics in CT images were studied using a Toshiba phantom containing five circular regions of differing CT# [−1000, −100,100,150,350] (HU) and a Toshiba Aquilion One CT scanner (this should be applicable to other scanners and can be easily tested in a similar way). As shown in Figure 8, six regions are considered in the phantom: a region in the background (with CT# of 0 HU) and the five regions inside the circles. Scanning was performed with eight different doses controlled by changing the tube current [5,10,25,50,100,150,200,250] (mAs) and with a fixed peak voltage of 120 kV. The noise distribution in each region was compared with white Gaussian additive noise. As can be seen in Figure 9, the noise distribution matches white Gaussian noise with high accuracy, provided that the dose is sufficiently high, that is, greater than 50 mAs, as in our experiments. The noise distribution of four selected X-ray tube currents is shown in Figure 9.  Figure 10 summarizes the noise variance changes for each region with different CT#'s as a function of the dose. When tube current is less than 10 mA, the electronic noise dominates the photon fluctuations causing the measured CT# to be inaccurate. Moreover, the noise variances of the six regions differ, even for the same tube currents. More realistically, we also studied the noise statistics of soft tissue surrounding the lung in a commercially available adult thoracic anthropomorphic phantom (Lungman, Kyoto Kakagu, Japan). These pixels have very close CT# values and almost the same radial locations. It was observed that the noise of these regions follows a Gaussian distribution with an acceptable accuracy. Two examples are shown in Figure 11. Based on these results, we assume that the noise in small neighborhoods of the image with similar CT# follows a white Gaussian distribution. This assumption enables patch based methods, such as BM3D and K-SVD, to use a Gaussian noise model in the similar patches of the image and to perform the denoising on each 3D stack independently.
